# Association of Arsenic with Adverse Pregnancy Outcomes/Infant Mortality: A Systematic Review and Meta-Analysis

**DOI:** 10.1289/ehp.1307894

**Published:** 2015-01-27

**Authors:** Reginald Quansah, Frederick Ato Armah, David Kofi Essumang, Isaac Luginaah, Edith Clarke, Kissinger Marfoh, Samuel Jerry Cobbina, Edward Nketiah-Amponsah, Proscovia Bazanya Namujju, Samuel Obiri, Mawuli Dzodzomenyo

**Affiliations:** 1 Centre for Environmental and Respiratory Health Research, Faculty of Medicine, University Of Oulu, Finland; 2 Department of Biological, Environmental & Occupational Health Sciences, School of Public Health, College of Health Sciences, University of Ghana, Legon, Accra, Ghana; 3 Environment, Health and Hazards Lab, Department of Geography, Western University, London, Ontario, Canada; 4 Environmental Health Group, Department of Chemistry, University of Cape Coast, Cape Coast, Ghana; 5 Department of Geography, Western University Canada, London, Ontario, Canada; 6 Ghana Health Service, Accra, Ghana; 7 Public Health Unit (Biostatistics), Korle-bu Teaching Hospital, Accra, Ghana; 8 School of the Environment, Jiangsu University, Jiangsu, China; 9 Department of Economics, University of Ghana, Legon, Ghana; 10 Department of Child, Adolescent and Adult Health, National Institute for Health and Welfare, Oulu, Finland; 11 School of Health Sciences, University of Tampere, Tampere, Finland; 12 Council for Scientific and Industrial Research, Accra, Ghana

## Abstract

**Background:**

Exposure to arsenic is one of the major global health problems, affecting > 300 million people worldwide, but arsenic’s effects on human reproduction are uncertain.

**Objectives:**

We conducted a systematic review and meta-analysis to examine the association between arsenic and adverse pregnancy outcomes/infant mortality.

**Methods:**

We searched PubMed and Ovid MEDLINE (from 1946 through July 2013) and EMBASE (from 1988 through July 2013) databases and the reference lists of reviews and relevant articles. Studies satisfying our *a priori* eligibility criteria were evaluated independently by two authors.

**Results:**

Our systematic search yielded 888 articles; of these, 23 were included in the systematic review. Sixteen provided sufficient data for our quantitative analysis. Arsenic in groundwater (≥ 50 μg/L) was associated with increased risk of spontaneous abortion (6 studies: OR = 1.98; 95% CI: 1.27, 3.10), stillbirth (9 studies: OR = 1.77; 95% CI: 1.32, 2.36), moderate risk of neonatal mortality (5 studies: OR = 1.51; 95% CI: 1.28, 1.78), and infant mortality (7 studies: OR = 1.35; 95% CI: 1.12, 1.62). Exposure to environmental arsenic was associated with a significant reduction in birth weight (4 studies: β = –53.2 g; 95% CI: –94.9, –11.4). There was paucity of evidence for low-to-moderate arsenic dose.

**Conclusions:**

Arsenic is associated with adverse pregnancy outcomes and infant mortality. The interpretation of the causal association is hampered by methodological challenges and limited number of studies on dose response. Exposure to arsenic continues to be a major global health issue, and we therefore advocate for high-quality prospective studies that include individual-level data to quantify the impact of arsenic on adverse pregnancy outcomes/infant mortality.

**Citation:**

Quansah R, Armah FA, Essumang DK, Luginaah I, Clarke E, Marfoh K, Cobbina SJ, Nketiah-Amponsah E, Namujju PB, Obiri S, Dzodzomenyo M. 2015. Association of arsenic with adverse pregnancy outcomes/infant mortality: a systematic review and meta-analysis. Environ Health Perspect 123:412–421; http://dx.doi.org/10.1289/ehp.1307894

## Introduction

Arsenic contamination of drinking water, air, food, and beverages is one of the major global health problems (Essumang 2009; Essumang et al. 2007; Hughes 2006; Navas-Acien and Nachman 2013; Obiri et al. 2010) that affect > 300 million people worldwide. This includes an estimated 13 million people in the United States and about 70 million people in Bangladesh (Murcott 2012). At concentrations > 50 μg/L, inorganic arsenic (iAs) has been associated with elevated risk of cancer (e.g., bladder, kidney, liver, lung, skin, prostate) (Ahamed et al. 2006b; McDonald et al. 2007; Mink et al. 2008; Steinmaus et al. 2000, 2003; Walvekar et al. 2007), cardiovascular diseases (Moon et al. 2013; Navas-Acien et al. 2005), high blood pressure (Abhyankar et al. 2012; Moon et al. 2013; Navas-Acien et al. 2005), anemia in pregnancy (Hopenhayn et al. 2006; Navas-Acien et al. 2006), mortality from respiratory diseases in both adults and children (Ahamed et al. 2006a; Ferreccio and Sancha 2006; Walvekar et al. 2007), diabetes in adults (Navas-Acien et al. 2006), and neurodevelopment problems (Hamadani et al. 2011). At concentrations around 10 μg/L, which is considered safe by the World Health Organization’s (WHO) provisional guideline (WHO 2011), iAs may still cause cancer in approximately 0.1–0.3% and increased systolic blood pressure in women 6 weeks postpartum (International Agency for Research on Cancer 2004; Kwok 2007). iAs easily crosses human and animal placenta and has been demonstrated to increase the risk of impaired fetal growth and infant mortality in laboratory animal studies (Navarro et al. 2004; Smith and Steinmaus 2009; Vahter 2009). Several epidemiologic studies (e.g., Cherry et al. 2010; Myers et al. 2010; Rahman et al. 2010) have examined the relation between arsenic and adverse pregnancy outcomes/infant mortality, and the findings are equivocal. Our understanding of arsenic exposure and adverse pregnancy outcomes is limited and, at best, fragmented. To our knowledge, no systematic review and/or meta-analysis has reported on the effect of arsenic on human pregnancy and infant health. Given the widespread low, moderate, and high arsenic exposure in the general population, an understanding of the impact of iAs on maternal and fetal health is relevant for public health policy.

To fill this gap, we conducted a systematic review and meta-analysis of epidemiologic studies to examine the association between arsenic exposure and the risk of spontaneous abortion, stillbirth, preterm delivery, birth weight, and neonatal/infant mortality.

## Methods

**Figure 1:**
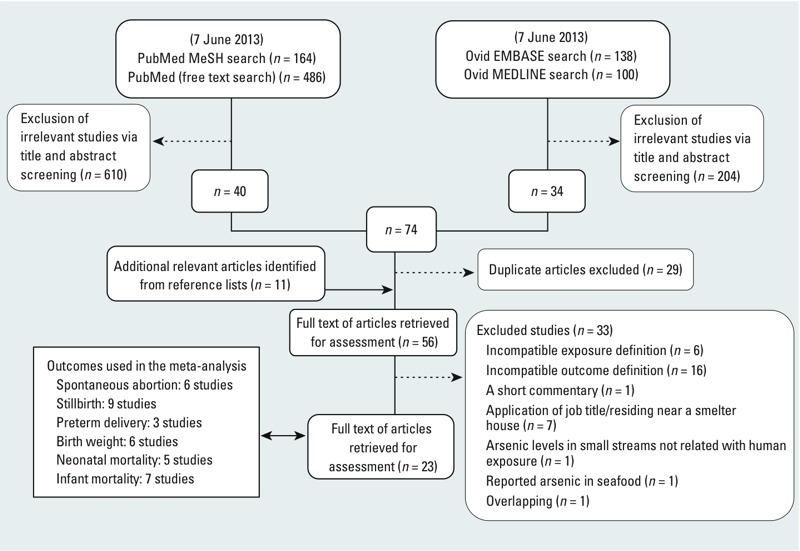
Study selection flow diagram.

*Search strategy and study selection*. This study was conducted according to the guidelines of the Preferred Reporting Items for Systematic Reviews and Meta-analysis (PRISMA) group (Moher et al. 2009). We searched PubMed (http://www.ncbi.nlm.nih.gov/pubmed) and Ovid MEDLINE (http://ovidsp.tx.ovid.com) (from 1946 through July 2013) and EMBASE (http://www.embase.com/login) (from 1988 through July 2013) databases (Figure 1), using the terms “arsenic,” “arsenicals,” “arsenite,” “arsenate” and “abortion, spontaneous,” “fetal mortality,” “preterm delivery,” “low birthweight,” “birthweight,” “infant mortality,” “neonatal mortality” (see Supplemental Material, “Search Strategy”). In addition, we searched the reference lists of reviews (Bloom et al. 2010; Smith and Steinmaus 2009; Vahter 2009) and potentially relevant articles. Two authors (R.Q. and F.A.A.) independently evaluated the articles. Studies that fulfilled the following *a priori* eligibility criteria were included if the study *a*) was an original study; *b*) was a cross-sectional, or a case–control or a cohort design; *c*) reported on any one or more of the following outcomes: spontaneous abortion, stillbirth, preterm delivery, birth weight, and neonatal/infant mortality; and *d*) presented data on arsenic exposure determined using environmental measures (arsenic in drinking water or airborne arsenic, or arsenic in soil), biomarkers, or indirect measures (e.g., residing in arsenic endemic area). Our exclusion criteria were *a*) a study was an experimental or a case report or a case series or a letter, *b*) a study was of arsenic compounds for which human exposure was unlikely [e.g., arsenic in roots of plants (Landgren 1996)], *c*) a study used job title or living close to a smelter house as surrogate for arsenic exposure, and *d*) a study did not include our relationships of interest.

If more than one report was published from the same study, the most recent study or the study using the best assessment of arsenic and/or outcome was included. For studies that reported estimates for more than one biomarker, the estimate for the most appropriate biomarker was preferred. The order of preference was as follows: nail > hair > urine. If a study provided estimates for water and a biomarker, the estimate from the latter was used.

*Data extraction and quality assessment*. Most relevant characteristics of eligible studies including study design, study size, location and country of study, method of arsenic assessment, exposure marker for arsenic, exposure contrast, exposure dose, type of adverse pregnancy/infant mortality and their definitions, year of publication, year of data collection, adjustment for adequate confounders, and study results (i.e., measures of association) were recorded in a standard data extraction form (Quansah and Jaakkola 2010) independently by two authors (R.Q. and F.A.A.). Any discrepancies were resolved by consensus. R.Q. and F.A.A. applied the Newcastle–Ottawa Scale (Wells et al. 2009) for observational studies to assess quality of eligible studies, with the maximum score of 9. Studies scoring ≥ 7 were categorized as high quality (see Supplemental Material, Tables S1 and S2).

*Statistical methods*. First, odds ratio (OR) or relative risk (RR) and their 95% confidence intervals (CIs) were derived or abstracted from eligible studies. Almost all the studies presented ORs and their 95% CIs; therefore, we used ORs in our analysis. One study (Ahmad et al. 2001) presented adjusted ORs and exact *p*-values but not the 95% CI, so we calculated the 95% CI from the *p*-values following Borenstein et al. (2009). Two studies (Guan et al. 2012; Rahman et al. 2007) presented RRs, and these were converted to ORs (Zhang and Yu 1998). Some relevant studies presented ORs for more than two exposure levels for the outcomes; therefore, in the meta-analysis we calculated summary ORs comparing our outcomes of interest (adverse pregnancy outcomes/infant mortality) in the highest (exposed group) and lowest (reference group) arsenic exposure categories presented in the studies. The exposed groups were heterogeneous and consisted of populations exposed to arsenic dose above the WHO guideline (i.e., > 10 μg/L) (WHO 2011). We separated the groups into high dose (exposed to ≥ 50 μg/L) and low-to-moderate dose (exposed to < 50 μg/L) for further analysis (explained below).

We applied the random-effects model (Borenstein et al. 2009) because we anticipated heterogeneity in the study-specific estimates. In the forest plots, we presented summary ORs of the random-effects model. Heterogeneity was computed using the *Q* (*p* < 0.1 considered significant), and *I*^2^-statistics (*I*^2^-statistic > 50% indicates high, 25–50% moderate, and < 25% low heterogeneity). We examined the influence of various characteristics on the study-specific effect estimates by stratifying the analysis by *a*) arsenic dose [i.e., high arsenic dose (> 50 μg/L) vs. low-to-moderate arsenic dose (< 50 μg/L)] and *b*) arsenic measured using individual data versus group data. We also performed a series of sensitivity analyses. First, we investigated the relative influence of each study on the summary OR by omitting each study one at a time. None of the studies had substantial influence on the summary ORs for our relations of interest and this finding was not reported. Second, we restricted the analysis to high-quality studies, prospective cohort studies, and studies adjusting for potential adequate confounders (see Supplemental Material, Table S3) documented in the literature (e.g., Di Mario et al. 2007; Ghosh 2012; Kramer 1987, 2003; McClure et al. 2006; Moss et al. 2002; Shah et al. 2011). We also presented dose response for studies with at least three exposure levels graphically. Publication bias was explored with funnel plots. The trim and fill method was used to assess the potential impact of missing studies in the funnel plot. Statistical analysis was performed using STATA software version 9 (StataCorp, College Station, TX, USA).

## Results

*Study characteristics*. Our systematic literature search strategy is shown in Figure 1. A total of 888 studies were retrieved, from which 56 studies were reviewed in depth. Twenty-three studies fulfilled our *a priori* inclusion criteria (Table 1) for this systematic review. Data from 16 studies were included in our quantitative analysis (see Supplemental Material, Tables S4 and S5). Thirty-three studies were excluded for various reasons (see Supplemental Material, Table S6). We had no data from the authors of two studies on birth weight (Fei et al. 2013; Guan et al. 2012) to calculate 95% CIs, so we included the studies only in our qualitative analysis. Five studies (Ahamed et al. 2006b; Chakraborti et al. 2003; Mukherjee et al. 2005; Rahman et al. 2005; Sen and Chaudhuri 2008) did not control for potential confounders, and they were also included in our qualitative analysis. Five studies (Cherry et al. 2008, 2010; Hopenhayn-Rich et al. 2000; Milton et al. 2005; Myers et al. 2010) were ecological retrospective cohort designs. Two studies were ecological case–control designs (Aschengrau et al. 1989; Ihrig et al. 1998). Only 1 (Rahman et al. 2007) of the 5 prospective cohort designs was an ecological study. Seven of the 10 cross-sectional designs were ecological studies. Nine studies were conducted in Bangladesh, 5 in India, 3 in China, 2 in Chile, 1 in Taiwan, and 3 in the United States. Twenty-two studies were conducted in populations exposed to arsenic in drinking water. Of these, 6 applied biomarkers including urine, maternal/cord/placenta blood, hair, and nail; and the remaining studies measured arsenic dose at the region/village/household level. Ihrig et al. (1998) measured arsenic dose in airborne emissions. Huyck et al. (2007) measured maternal hair arsenic dose at first prenatal visit, maternal hair arsenic dose at birth, and maternal nail arsenic dose at first prenatal visit, but the estimates of the latter biomarker was considered suitable and included in the meta-analysis. There were 10 reports on spontaneous abortion, 14 on stillbirth, 3 on preterm delivery, 6 on birth weight, 5 on neonatal mortality, and 7 on infant mortality. Eligible studies applied either questionnaire/interview or hospital/medical records or national registers to ascertain information on the outcomes of interest. Most of the studies (Table 1) scored low on the Newcastle–Ottawa Scale for several reasons, including bias associated with selection of study population, measurement of arsenic exposure, lack of individual arsenic data, inappropriate definition of cases/controls, inappropriate comparable reference, and a lack of adequate adjustment for potential confounders (Table 1).

**Table 1:** Characteristics of studies included in the systematic review and meta-analysis.

Sources (study design)	Location	Study population	Arsenic concentration			Outcome studied	Confounders adjusted for	Total score on NOS
			Marker for exposure	Exposure contrast	Range, median, or mean			
Fei et al. 2013^*a*^^,^^*b*^ (PCO)	New Hamsphire (USA)	133 pregnant women	Arsenic levels in urine	NA	Not reported	Birth weight	Infant sex, maternal age, gestational age	7/9
Guan et al. 2012^*b*^^,^^*c*^ (CS)	Dalian (China)	125 mother–infant pairs	Arsenic levels in maternal and cord blood	Arsenic-affected area (590 μg/L) vs. arsenic-free area	Not reported	Birth weight	Maternal age, BMI, parity, gestational age at delivery, maternal education, maternal secondhand smoke exposure, infant sex	5/9
Cherry et al. 2010^*c*^^,^^*d*^ (RCO)	Gonoshasthaya Kendra villages (Bangladesh)	934 infant mortality occurring in designated area, 2001–2003	Arsenic levels in tube-well water	≥ 50 μg/L vs. < 10 μg/L	0.05–166 μg/L	Infant mortality	First pregnancies, others with no formal education, mothers designated as destitute	7/9
Myers et al. 2010^*c*^^,^^*d*^ (RCO)	Bayingnormen (Mongolia, China)	9,890 singleton deliveries of mothers	Arsenic levels in tube-well water	> 50 μg/L vs. ≤ 50 μg/L	UD–1,200 μg/L	Birth weight, preterm delivery, stillbirth, and neonatal mortality	Maternal age, gravidity, infant sex for the analysis of birth weight and maternal age, gravidity, infant sex adequacy for the analysis of preterm delivery, stillbirth, and neonatal mortality	7/9
Rahman et al. 2010^*b*^^,^^*c*^ (PCO)	Matlab district (Bangladesh)	2,924 pregnant women	Arsenic levels in urine	249–1,253 μg/L vs. < 33 μg/L (spontaneous abortion)268–2,019 μg/L vs. < 38 μg/L (stillbirth)268–2,019 vs. < 38 μg/L (infant mortality)	UD–1,253 μg/L	Spontaneous abortion, stillbirth, infant mortality	No significant confounder was found	7/9
Rahman et al. 2009^*b*^^,^^*c*^ (PCO)	Matlab (Bangladesh)	1,578 women with single births	Arsenic concentrations in urine	≥ 100 μg/L vs. < 100 μg/L	6–978 μg/L	Birth weight	Asset score, BMI, height, age, education, season, gestational age at birth, sex of infant	8/9
Cherry et al. 2008^*c*^^,^^*d*^ (RCO)	Villages in 13 subdistricts (Bangladesh)	30,984 pregnancies and outcomes	Average arsenic concentrations in hand-pumped well water	≥ 50 μg/L vs. < 0.10 μg/L	UD–81 μg/L	Stillbirth	Age, sex, previous pregnancy, previous stillbirth, low socioeconomic status, maternal education, paternal education, maternal smoking, mother high BP, mother edema, gestational age, birth weight, home delivery	8/9
Sen and Chaudhuri 2008^*c*^^,^^*d*^^,^^*e*^ (CS)	Villages located in North 24 Parganas district (states of West Bengal)	Pregnancy outcomes of 240 married women	Arsenic levels in tube-well water	600 μg/L vs. < 10 μg/L	10–600 μg/L	Spontaneous abortion and stillbirth	None	2/9
Huyck et al. 2007^*b*^^,^^*c*^ (PCO)	42 villages in Sirajdikhan Upakila of Munshigani district (Bangladesh)	49 women ≥ 18 years of age	Arsenic levels in maternal hair at first visit	≥ 2.70 μg/g vs. < 0.28 μg/g	0.14–3.28 μg/g	Birth weight	Gestational age at first prenatal visit, maternal weight gain, birth gestational age, and activity level during pregnancy	7/9
Rahman et al. 2007^*c*^^,^^*d*^ (PCO)	Matlab (Bangladesh)	29,134 pregnancies identified by the HDSS in 1991–2000	Arsenic levels in tube-well water	≥ 409 μg/L vs. < 10 μg/L	Median, 224 μg/L	Fetal loss, infant mortality, neonatal	Age, parity, education, and socioeconomic status	7/9
Ahamed et al. 2006a, 2006b^*c*^^,^^*d*^^,^^*e*^ (CS)	Eruani village (Bangladesh)	56 pregnancy outcomes in women of reproductive age	Arsenic levels in tube-well water	Exposed area (501–1,200 μg/L) vs. control area	501–1,200 μg/L	Spontaneous abortion and stillbirth	None	1/9
von Ehrenstein et al. 2006^*c,d*^ (CS)	21 villages in West Bengali (south 24-Parganas district) (India)	202 married women, 20–40 years of age	Arsenic levels in tube-well water	≥ 200 μg/L vs. < 50 μg/L	Mean = 101.7 μg/L	Spontaneous abortion, stillbirth, neonatal mortality, infant mortality	Mother’s age at child’s birth, BMI, maternal education, education of the head of the household, and type of housing material	3/9
Milton et al. 2005^*c*^^,^^*d*^ (CS)	29 villages in Comilla district, 2 villages in the Chandpur district, 43 villages in the Chaudanga district (Bangladesh)	533 ever-married women, 15–49 years of age	Arsenic levels in tube-well water	> 50 μg/L vs. ≤ 50 μg/L	UD–1,710 μg/L	Spontaneous abortion, stillbirth, and neonatal mortality	Height, history of hypertension and diabetes, and age at first pregnancy for neonatal mortality	3/9
Mukherjee et al. 2005^*c*^^,^^*d*^^,^^*e*^ (CS)	Murshidabad (West Bengal, India)	17 married women of reproductive age (18–40 years) with at least 1 pregnancy	Arsenic levels in drinking water	Exposed area (401–1,474 μg/L) vs. nonexposed area (< 3 μg/L)	401–1,474 μg/L	Spontaneous abortion and stillbirth	None	1/9
Rahman et al. 2005^*c*^^,^^*d*^^,^^*e*^ (CS)	Jalangi block (India)	13 married women of reproductive age (18–40 years)	Arsenic levels in drinking water	Women in exposed areas (501–1,474 μg/L) vs. women in control area (< 10 μg/L)	Not reported	Spontaneous abortion and stillbirth	None	1/9
Chakraborti et al. 2003^*c*^^,^^*d*^^,^^*e*^ (CS)	Semria Ojha Patti village of Ara (Bhoipur, India)	16 women	Arsenic levels in tubes-well water	463–1,025 μg/L vs. 7–39 μg/L	7–1,025 μg/L	Stillbirth	None	1/9
Guo et al. 2003^*a*^^,^^*d*^ (CS)	Villages in Wuyan County (Inner Mongolia, China)	224 women	Arsenic levels in well water	Exposed area (43 μg/L) vs. nonexposed area (9.6 μg/L)	Not reported	Spontaneous abortion	Sex, age, smoking and alcohol consumption	3/9
Hopenhayn et al. 2003^*a*^^,^^*d*^ (PCO)	Antofagasta and Valparaiso (Chile)	844 singleton mothers 18–45 years of age	Arsenic levels in water	40 μg/L vs. < 1 μg/L	32.9–52.7 μg/L	Birth weight	Location, calendar time, arsenic exposure	6/9
Yang et al. 2003^*a*^^,^^*d*^ (RCO)	18 villages in 4 township in Lanyang Basin (Taiwan)	18,259 singleton births	High arsenic–exposed community used as a surrogate	Exposed area (UD–3,590 μg/L) vs. nonexposed area	UD–3.59 ppm	Preterm delivery, birth weight	Maternal age, marital status, maternal education, infant sex	6/9
Ahmad et al. 2001^*c*^^,^^*d*^ (CS)	Village of Samta in thana Sharsha, Jessore district; village of Katiarchar in Sadar thana, Kishorgonj district (Bangladesh)	192 married women of reproductive age (15–49 years)	Arsenic levels in tube-well water	> 50 μg/L vs. ≤ 0.2 μg/L	200–450 μg/L	Spontaneous abortion, stillbirth, and preterm birth	Socioeconomic status, education, and age at marriage	3/9
Hopenhayn-Rich et al. 2000^*c*^^,^^*d*^ (RCO)	Antofagasta and Valparaiso (Chile)	Mortality of infants, 1950–1996	Arsenic levels in public water	> 50 vs. 5 μg/L	40–860 μg/L	Fetal mortality, neonatal mortality,	Location, calendar time, arsenic exposure	6/9
Ihrig et al. 1998^*c*^^,^^*d*^ (C‑;C)	Bryan, TX (USA)	119 case babies, and 267 control babies	Arsenic levels estimated from airborne emissions	> 100 vs. 0 ng/m^3^	Not reported	Stillbirths	Maternal age, race/ethnicity, parity, income group, exposure as a categorical variable, and exposure–race/ethnicity interaction	7/9
Aschengrau et al. 1989 (C-C)^*a*^^,^^*d*^	Boston, MA (USA)	286 cases, 1,391 controls	Arsenic levels in public drinking water	1.4–1.9 μg/L vs. UD	UD–19 μg/L	Spontaneous abortion	Water source, maternal age, educational level, history of prior spontaneous abortion	7/9
Abbreviations: BMI, body mass index; BP, blood pressure; C-C, case–control study; CS, cross-sectional study; HDSS, health and demographic surveillance system; NA, not applicable; NOS, Newcastle–Ottawa Scale; PCO, prospective cohort study; RCO, retrospective cohort study; UD, undetected. ^***a***^Studies examining low to moderate arsenic dose in the general population. ^***b***^Studies examining high arsenic dose in the general population. ^***c***^Studies applying biomarkers/individual-level data. ^***d***^Studies applying group/ecological data. ^***e***^Studies that did not control for potential confounders.								

**Figure 2:**
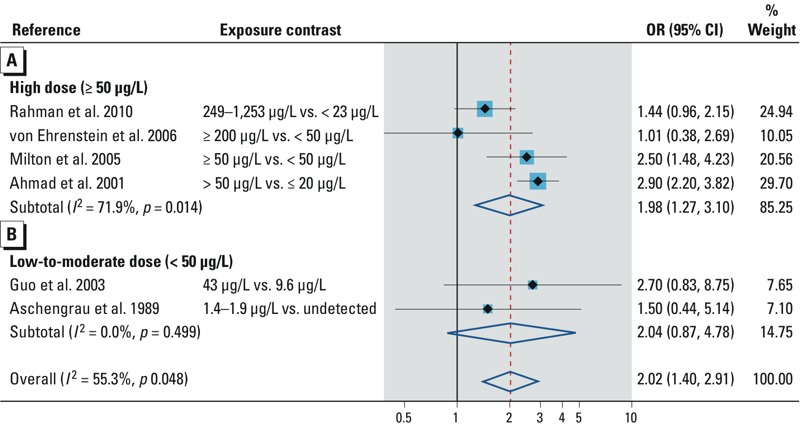
Forest plot for the relation between arsenic exposure and the risk of spontaneous abortion, assessed by (*A*) high arsenic dose and (*B*) low to moderate arsenic dose.

**Figure 3:**
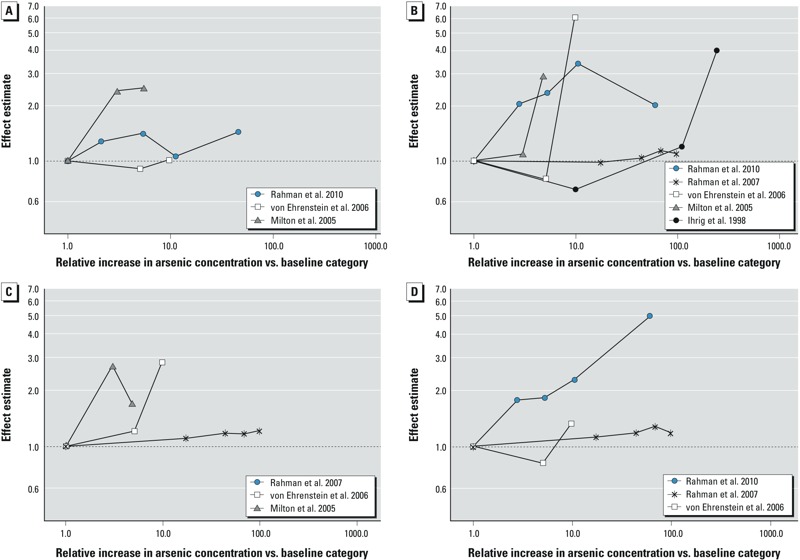
Plots of dose–response relations for arsenic and (*A*) spontaneous abortion, (*B*) stillbirth, (*C*) neonatal mortality, and (*D*) infant mortality in the general population.

*Arsenic exposure in the general population*. Spontaneous abortion. In all 10 studies that examined the association with spontaneous abortion, 4 were excluded from our quantitative analysis because the authors did not control for potential confounders. Sen and Chaudhuri (2008) studied outcome of pregnancy in 240 married women. In women with the highest concentrations of arsenic in drinking water (501–1,200 μg/L), there was an increase in spontaneous abortion. A similar observation was noted by Ahamed et al. (2006a), Mukherjee et al. (2005), and Rahman et al. (2005). Six studies provided data for our quantitative analysis (see Supplemental Material, Table S4). All the studies reported ORs. Summary OR in populations exposed to high arsenic dose (> 50 μg/L) in groundwater showed increased association (OR = 1.98; 95% CI: 1.27, 3.10) (Figure 2A). Our finding in populations exposed to low to moderate arsenic in groundwater (Guo et al. 2003) or in public tap water (Aschengrau et al. 1989) was inconclusive (Figure 2B). Overall, the summary OR was 2.02 (95% CI: 1.40, 2.91). The direction and magnitude of the association persisted in studies applying biomarkers/individual arsenic data, prospective studies, studies adjusting for adequate potential confounders, and high-quality studies (Table 2). Figure 3A shows the dose–response relation of arsenic in drinking water and spontaneous abortion. The risk trend was not consistent across the studies. A funnel plot suggested influence of publication bias (see Supplemental Material, Figure S1A), and an adjustment with the trim and fill method did not change the strength of the overall summary OR (Table 2).

**Table 2:** Summary OR for the relation between arsenic and the risk of adverse pregnancy/infant mortality and stratified/sensitivity analysis according to the study characteristics.

Analysis	Spontaneous abortion		Stillbirth		Neonatal mortality		Infant mortality	
	Random-effects model OR (95% CI)	Heterogeneity statistics Q (*n*)‑statistics, *I*^2^‑index (%), *p*‑value	Random-effects model OR (95% CI)	Heterogeneity statistics Q (*n*)‑statistics, *I*^2^‑index (%), *p*‑value	Random-effects model OR (95% CI)	Heterogeneity statistics Q (*n*)‑statistics, *I*^2^‑index (%), *p*‑value	Random-effects model OR (95% CI)	Heterogeneity statistics Q (*n*)‑statistics, *I*^2^‑index (%), *p*‑value
Summary OR	2.02 (1.40, 2.91)	11.2 (6), 55.3, 0.048	1.84 (1.38, 2.45)	38.40 (9), 79.2, 0.000	1.51 (1.28, 1.78)	5.34 (5), 25.1, 0.254	1.35 (1.12, 1.62)	8.31 (7), 30.4, 0.216
Stratified analysis
Assessment of arsenic exposure
Individual data/biomarker	2.20 (1.04, 3.46)	7.96 (3), 74.9, 0.019	1.96 (1.17, 3.29)	19.91 (5), 79.9, 0.001	1.30 (1.00, 1.67)	4.78 (2), 16.4, 0.274	1.74 (0.92, 3.28)	6.02 (3), 66.8, 0.049
Group data	1.51 (0.79, 2.87)	1.59 (3), 0.0, 0.951	1.79 (1.29, 2.48)	5.46 (4), 45.1, 0.141	1.59 (1.43, 1.77)	1.75 (3), 0.0, 0.416	1.32 (1.08, 1.60)	2.44 (4), 0.0, 0.486
Sensitivity analysis
Prospective cohort studies^*a*^	1.45 (0.99, 2.12)	0.66 (2), 0.0, 0.951	1.13 (0.98, 1.30)	0.67 (2), 0.0, 0.412	1.21 (0.98, 1.50)^*a*^		2.12 (0.53, 8.42)	4.84 (2), 79.3, 0.028
Studies adjusting for potential confounders	1.72 (1.25, 2.37)	4.43 (5), 9.7, 0.351	1.85 (1.22, 2.82)	18.06 (7), 66.8, 0.005	1.53 (1.11, 2.10)	4.63 (4), 35.2, 0.201	1.65 (1.01, 2.47)	8.39 (5), 52.3, 0.078
High-quality studies (> 7 on NOS)	1.45 (0.99,1.12)	1.65 (3), 0.0, 0.438	1.28 (0.98, 1.67)	4.27 (4), 29.7, 0.234	1.49 (0.92, 2.31)	2.57 (2), 61.1, 0.109	1.41 (1.04,1.92)	7.55 (4), 60.2, 0.056
Impact of missing studies on overall summary OR
By trim and fill method	2.02 (1.20, 2.91)	55.3 (6), 55.3, 0.048	1.43 (1.11, 1.85)	58.06 (13), 79.33, 0.000	1.47 (1.27, 1.71)	7.15 (7), 2.15, 0.307	1.22 (0.98, 1.53)	8.62 (10), 30.0, 0.017
Abbreviations: NOS, Newcastle–Ottawa Scale; Q(*n*), *n*: number of studies.^***a***^One prospective study reported on neonatal mortality.								

**Figure 4:**
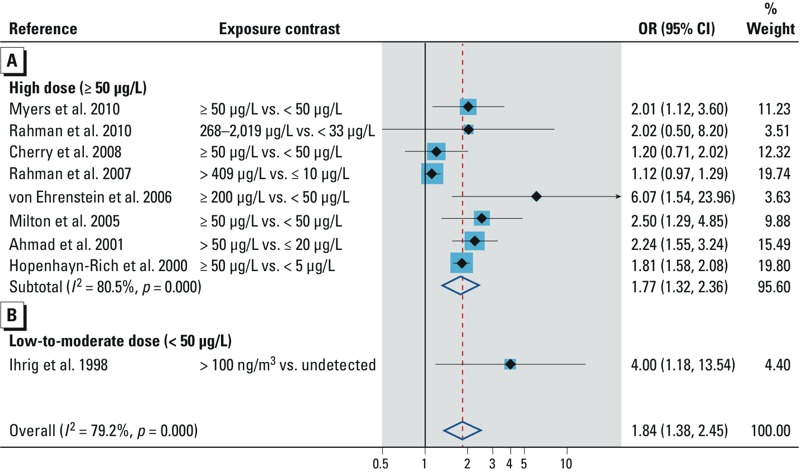
Forest plot for the relation between arsenic exposure and the risk of stillbirth, assessed by (*A*) high arsenic dose and (*B*) low to moderate arsenic dose.

Stillbirth. Of the 14 studies reporting association with stillbirth, 5 (Ahamed et al. 2006a; Chakraborti et al. 2003; Mukherjee et al. 2005; Rahman et al. 2005; Sen and Chaudhuri 2008) were excluded from our quantitative analysis because the authors did not control for potential confounders. All 5 studies observed an increase in stillbirth in women who had the highest concentrations of arsenic in their drinking water. Nine studies examining the association between environmental arsenic and stillbirth provided data for our quantitative analysis (see Supplemental Material, Table S4). Two studies (Hopenhayn-Rich et al. 2000; Rahman et al. 2007) reported RRs. Arsenic was measured in groundwater in 8 studies and in air in 1 study. Summary OR in populations exposed to high arsenic dose (> 50 μg/L) in groundwater was increased (OR = 1.77; 95% CI: 1.32, 2.36; Figure 4A). Only 1 study (Ihrig et al. 1998) was conducted in a population exposed to low-to-moderate arsenic dose (Figure 4B). The overall summary OR for environmental arsenic was 1.84 (95% CI: 1.38, 2.45). In subgroup/sensitivity analyses, the risk of stillbirth was increased in studies applying biomarkers/individual arsenic data, studies using group data on arsenic, studies adjusting for adequate potential confounders, prospective studies, and high-quality studies (Table 2). Five studies reported a dose–response relationship between environmental arsenic and stillbirth (Figure 3B). Risk trend was consistent in 2 studies in high arsenic dose area (Milton et al. 2005; Rahman et al. 2010), but this trend was not obvious in the other studies. A funnel plot suggested influence of small positive studies (see Supplemental Material, Figure S1B). The trim and fill method for adjustment of publication bias imputed 4 studies, and, as expected, the strength of the summary OR was attenuated but remained statistically significant (Table 2).

**Figure 5:**
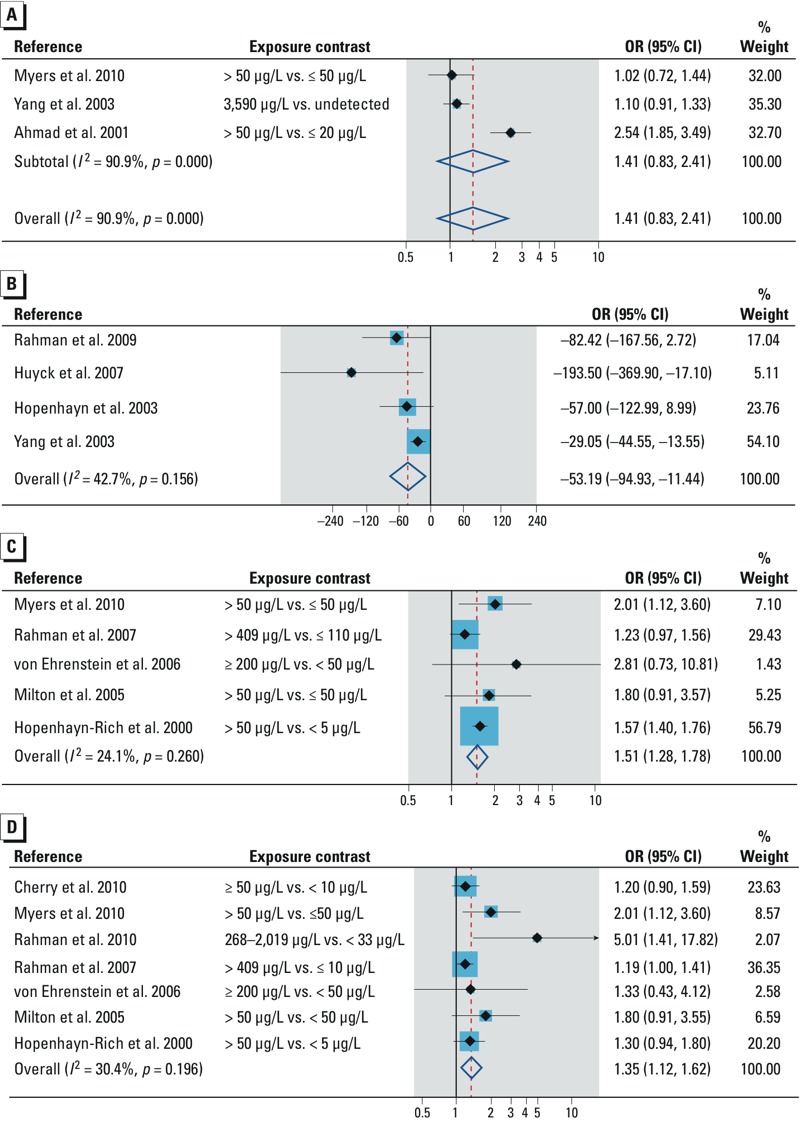
Forest plot for the relation between arsenic exposure and (*A*) preterm delivery, (*B*) birth weight, (*C*) neonatal mortality, and (*D*) infant mortality.

Preterm delivery. In all, three studies (Ahmad et al. 2001; Myers et al. 2010; Yang et al. 2003) investigated the relation between arsenic exposure and preterm delivery (see Supplemental Material, Table S4). They all reported ORs and were conducted in populations exposed to high arsenic dose in groundwater. The finding of the summary OR was inconclusive (OR = 1.41; 95% CI: 0.83, 2.41) (Figure 5A).

Birth weight. The association between arsenic and birth weight was examined in six studies. Two studies did not provide sufficient quantitative data for the meta-analysis. Fei et al. (2013) measured arsenic dose in maternal urine (UAs) and observed an inverse dose response (coefficient: β = –1.3) between UAs and birth weight. Guan et al. (2012) also observed that newborns of mothers whose UAs was > 5.30 μg/L weighed on average 0.22 kg less than those of mothers whose UAs was ≤ 5.30 μg/L. Four studies (Hopenhayn et al. 2003; Huyck et al. 2007; Rahman et al. 2009; Yang et al. 2003) provided regression coefficient and standard errors for our quantitative analysis (see Supplemental Material, Table S4). Environmental arsenic shows a significant reduction in birth weight (–53.2 g; 95% CI: –94.9, –11.4) (Figure 5B).

Neonatal mortality. Five studies examined neonatal mortality (see Supplemental Material, Table S5), of which two (Hopenhayn-Rich et al. 2000; Rahman et al. 2007) reported RRs. All the studies were conducted in populations exposed to high arsenic dose in groundwater. The overall summary OR was 1.51 (95% CI: 1.28, 1.78) (Figure 5C). The direction of association did not change in studies applying biomarkers/individual arsenic data and in studies adjusting for adequate potential confounders (Table 2). Rahman et al. (2007) reported on this relation. Dose response was examined in three studies (Figure 3C.) A consistent dose–response trend was observed by von Ehrenstein et al. (2006), but the risk trend was inconsistent in studies by Milton et al. (2005) and Rahman et al. (2007). Evidence of publication bias was observed in the funnel plot (see Supplemental Material, Figure S1C). The trim and fill method imputed two studies, and the strength of association was reduced marginally (Table 2).

Infant mortality. Arsenic and infant mortality was investigated in seven studies (see Supplemental Material, Table S5), with two (Hopenhayn-Rich et al. 2000; Rahman et al. 2007) reporting RRs. The studies were conducted in populations exposed to high arsenic dose in groundwater. Summary OR was 1.35 (95% CI: 1.12, 1.62) (Figure 5D). Compared with that of the overall summary OR, the association was slightly elevated in studies applying biomarkers/individual arsenic data, studies adjusting for adequate potential confounders, and high-quality studies, but marginally reduced in studies using group data on arsenic (Table 2). Our findings from two prospective studies were inconclusive. Among three studies examining dose response (Figure 3D), a consistent risk trend was observed by Rahman et al. (2010), but the risk trend was not consistent in studies by Rahman et al. (2007) and von Ehrenstein et al. (2006). A funnel plot showed evidence of asymmetry, suggesting influence of small positive studies (see Supplemental Material, Figure S1D). As expected, the strength of association was attenuated with the trim and fill method, and three missing studies were imputed (Table 2).

## Discussion

This is the first systematic review and meta-analysis on the association between iAs exposure and adverse pregnancy outcomes/infant mortality. We found positive associations of arsenic with spontaneous abortion, stillbirth, birth weight, and neonatal and infant mortality. These findings are important to many countries around the globe where pregnant women and infants continue to be exposed to low through moderate to high arsenic dose in different media (e.g., drinking water, air, food, beverages).

*Validity issues*. Our study has a number of strengths. We searched several databases including reference lists of reviews and relevant studies. Two authors independently checked the eligibility of the studies according to a predefined set of criteria. We followed systematically the guideline of PRISMA (Moher et al. 2009).

Because the upper limits of arsenic exposure differed among studies, we studied the effect in populations in low-to-moderate arsenic areas (i.e., < 50 μg/L) separately from the effect in populations in high arsenic areas (i.e., ≥ 50 μg/L). In studies of spontaneous abortion and stillbirth, the findings were inconclusive.

We excluded from our quantitative analysis small ecological studies that did not adjust for potential confounders (Altman 1994; Turner et al. 2013). Also, in considering our core and additional confounders various studies should have adjusted for, we followed recommendations in the literature (e.g., Di Mario et al. 2007; Ghosh 2012; Kramer 1987, 2003; Kumar 2011; McClure et al. 2006; Moss et al. 2002; Shah et al. 2011).

While acknowledging the importance of our findings, we note a number of limitations. First, the use of summary scores to identify high-quality studies in Newcastle–Ottawa Scale is a bit problematic. A risk of bias tool that applies a domain-based evaluation may allow one to explore the influence of each domain on the overall summary effect estimate (Higgins and Green 2011; National Research Council 2004). Second, a well-designed study may be categorized as low quality because the authors failed to provide detail information in the publication. Finally, some items of the Newcastle–Ottawa Scale such as representativeness of study cohort with respect to community and duration of follow-up do not belong to the risk of bias tools (Deeks et al. 2003; National Research Council 2004; Sanderson et al. 2007). Thus, the interpretation of how well a study does on the Newcastle–Ottawa Scale in our study should be done with caution. Inclusion of ecological studies in our review may lead to underestimation of our observed associations. Also the studies incorporated in this meta-analysis were different with regard to exposure levels in the reference groups. However, in computing the overall summary OR from the different studies, we made an implicit assumption that any differences in exposure levels in the reference groups will not have much influence on our summary OR.

We observed substantial heterogeneity in the study-specific estimates for studies on spontaneous abortion and stillbirth, and moderate heterogeneity for studies on neonatal and infant mortality. In stratified analysis, heterogeneity persisted in studies applying biomarkers for the association with spontaneous abortion, stillbirth, and infant mortality. In sensitivity analyses, heterogeneity persisted in prospective studies on infant mortality, studies on stillbirth and infant mortality that have controlled for adequate potential confounders, and high-quality studies on neonatal and infant mortality. The original studies also applied different exposure assessment methods and incorporated different exposure contrasts, thus making it difficult to relate any exposure increase to change in birth weight. Differences in responses to arsenic exposure may also exist across study populations (Abhyankar et al. 2012; Concha et al. 2002; Hopenhayn-Rich et al. 1998), and these could be potential sources of the observed heterogeneity. We lacked data on these factors and we also did not have sufficient data from the original studies to elaborate further the reasons for the heterogeneity. We applied the trim and fill method to examine the impact of publication bias on our overall summary OR, and the summary OR was slightly reduced for stillbirth, neonatal mortality, and infant mortality, suggesting that publication bias is not an explanation of our observed associations. Nonetheless, the trim and fill method performs poorly in the presence of substantial heterogeneity, so the influence of publication bias on the observed associations cannot be ruled out.

Our findings, however, should be interpreted in the light of limitations inherent in the original studies. Some studies failed to adjust for appropriate potential confounders of adverse pregnancy outcomes/infant mortality and could not establish the independent role of arsenic. Although few studies adjusted for proxies of socioeconomic status, only one study considered access and utilization of prenatal care. This is an important socioeconomic factor to be considered in the studies of stillbirth and neonatal/infant mortality (Kiely et al. 1985; Ronsmans et al. 2004; Shah et al. 2011). Exposure assessment was also a major challenge in the studies. Three studies measured arsenic contents in urine (Fei et al. 2013; Rahman et al. 2007, 2010). One study measured arsenic content in blood (Guan et al. 2012), and Huyck et al. (2007) measured arsenic content in hair. Arsenic content in urine/blood is a marker of current exposure, whereas information on chronic exposure can be obtained from arsenic content in hair or finger/toe nails. The remaining studies applied ecological measures. Questionnaires were administered in most studies, but data on water consumption pattern (i.e., the frequency and quantity of water intake) were not reported. Lack of individual data may result in measurement error with underestimation of the true effect. Many of the studies were cross-sectional in design, precluding temporality. Although few studies have collected data on our outcomes of interest from medical records/established registers, most studies have relied on maternal recall. Methods applied in collecting data on spontaneous abortion were not sensitive enough to detect events occurring in early pregnancy. Thus, the fetal and infant health effect of arsenic observed in our study may have been substantially underestimated.

*Comparison with previous studies*. Only two qualitative reviews were available on this subject. In the first study, Smith and Steinmaus (2009) examined the effects of arsenic and chromium in drinking water on low birth weight and infant mortality. The authors identified 10 studies and failed to reach any conclusion. In the second study, Bloom et al. (2010) examined the association of spontaneous abortion with arsenic in drinking water. The authors also identified 9 studies and concluded that chronic exposure to arsenic was associated with spontaneous abortion. In the present study, we observed excess risk of 102% for spontaneous abortion, 84% for stillbirth, 51% for neonatal mortality, 35% for infant mortality, and a 53-g reduction in birth weight. The magnitude of association persisted in studies applying biomarkers, studies using aggregate data on arsenic exposure, studies adjusting for adequate potential confounders, and high-quality studies. From the global public health point of view, the observed association is relevant considering the magnitude of the estimated effect and the extent of exposure to arsenic globally.

The precise biologic window of susceptibility of arsenic for adverse pregnancy outcomes is unknown (Vahter 2007, 2009). But arsenic exposure at different periods before or during pregnancy could cause a wide range of adverse pregnancy outcomes (Selevan et al. 2000). In laboratory animals, prenatal arsenic exposure causes spontaneous abortion by defective implantation or zygote development and aneuploidy, or through aberrant placental vasculogenesis and placental insufficiency (He et al. 2007; Navarro et al. 2004). Epidemiologic studies have also shown that arsenic causes oxidative stress, lipid peroxidation, interference of hormonal activities, and perturbation of DNA methylation, which may be associated with a wide range of adverse pregnancy outcomes through defective placentation and preeclampsia (Concha et al. 1998; Hood et al. 1988; Hu et al. 1998; Hughes 2002; Vahter 2007, 2009).

Our findings suggest that the effect of arsenic is strongest for spontaneous abortion. Although methylation is expected to have improved dramatically in the second trimester (Concha et al. 1998; Vahter 2007), at high arsenic dose (≥ 50 μg/L) observed in the populations included in our study, methylation is inhibited and the fetus blood plasma may essentially contain unmethylated arsenic and monomethylarsonic acid, which could threatened fetal survival and growth (Hall et al. 2013; Vahter 2007). Exposure to arsenic *in utero* and in early life may also pose a threat to infant survival (Hughes 2002; Vahter 2007). This observation has been noted in series of cohort studies conducted in the developing countries (Milton et al. 2005; Myers et al. 2010; Rahman et al. 2009, 2010).

Studies with the greatest weight in the meta-analyses did not provide data for the evaluation of dose–response trend. However, in the few studies that provided data, we observed inconsistent dose–response trend at high arsenic dose. The evidence was scarce for low to moderate arsenic dose and for studies evaluating preterm delivery.

## Conclusions

Our systematic review and meta-analysis found positive associations of arsenic exposure with spontaneous abortion, stillbirth, birth weight, and neonatal and infant mortality. However, the interpretation of causal association of high arsenic dose in drinking water is limited by methodological problems in the original studies and limited studies on dose response.

## Supplemental Material

(772 KB) PDF
