# Supplementary material for: Association of Arsenic with Adverse Pregnancy Outcomes/Infant Mortality: A Systematic Review and Meta-Analysis
Source: Environ Health Perspect. 2015 Jan 27;123(5):412–21. doi: 10.1289/ehp.1307894 (PMC4421764; doi:10.1289/ehp.1307894)
Supplement: (772 KB) PDF [file ehp.1307894.s001.508.pdf]

Environmental Health Perspectives doi:10.1289/ehp.1307894

Supplemental Material

Association of Arsenic with Adverse Pregnancy Outcomes–Infant Mortality: A Systematic Review and Meta-Analysis

Reginald Quansah, Frederick Ato Armah, David Kofi Essumang, Isaac Luginaah, Edith Clarke, Kissinger Marfoh, Samuel Jerry Cobbina, Edward Nketiah-Amponsah, Proscovia Bazanya Namujju, Samuel Obiri, and Mawuli Dzodzomenyo

Supplemental Material, Search strategy

Database: Ovid MEDLINE(R) <1946 to July 2013>

Search Strategy:

----

1 arsenic.ab,al,hw,kf,kw,ot,sh,ti,fs,tw. (18061)
2 arsenicals.ab,al,hw,kf,kw,ot,sh,ti,fs,tw. (6800)
3 arsenite.ab,al,hw,kf,kw,ot,sh,ti,fs,tw. (4102)
4 arsenate.ab,al,hw,kf,kw,ot,sh,ti,fs,tw. (3144)
5 spontaneous abortion.ab,al,hw,kf,kw,ot,sh,ti,fs,tw. (5275)
6 fetal mortality.ab,al,hw,kf,kw,ot,sh,ti,fs,tw. (1393)
7 preterm delivery.ab,al,hw,kf,kw,ot,sh,ti,fs,tw. (6024)
8 low birthweight.ab,al,hw,kf,kw,ot,sh,ti,fs,tw. (5437)
9 birth weight.ab,al,hw,kf,kw,ot,sh,ti,fs,tw. (65080)
10 infant mortality.ab,al,hw,kf,kw,ot,sh,ti,fs,tw. (27359)
11 neonatal mortality.ab,al,hw,kf,kw,ot,sh,ti,fs,tw. (4451)
12 1 or 2 or 3 or 4 (23599)
13 5 or 6 or 7 or 8 or 9 or 10 or 11 (98265)
14 12 and 13 (100)
15. from 14 keep 4,8-10,12-15,17-18,24,26-29,31,37-38,41,43,45,47-48,52-54,58,61-64,66-68,71,77-78,82-84 (40)
16. from 15 keep 4,8-10,12-15,17-18,24,26-29,31,37-38,41 (19)

Database: Ovid EMBASE < 1988 to July 2013>
Search Strategy:
----
1 arsenic.ab,al,hw,kf,kw,ot,sh,ti,fs,tw. (18061)
2 arsenicals.ab,al,hw,kf,kw,ot,sh,ti,fs,tw. (6800)
3 arsenite.ab,al,hw,kf,kw,ot,sh,ti,fs,tw. (4102)
4 arsenate.ab,al,hw,kf,kw,ot,sh,ti,fs,tw. (3144)
5 spontaneous abortion.ab,al,hw,kf,kw,ot,sh,ti,fs,tw. (5275)
6 fetal mortality.ab,al,hw,kf,kw,ot,sh,ti,fs,tw. (1393)
7 preterm delivery.ab,al,hw,kf,kw,ot,sh,ti,fs,tw. (6024)
8 low birthweight.ab,al,hw,kf,kw,ot,sh,ti,fs,tw. (5437)
9 birth weight.ab,al,hw,kf,kw,ot,sh,ti,fs,tw. (65080)
10 infant mortality.ab,al,hw,kf,kw,ot,sh,ti,fs,tw. (27359)
11 neonatal mortality.ab,al,hw,kf,kw,ot,sh,ti,fs,tw. (4451)
12 1 or 2 or 3 or 4 (23599)
13 5 or 6 or 7 or 8 or 9 or 10 or 11 (98265)
14 12 and 13 (138)
15. from 14 keep 4,8-10,12-15,17-18,24,26-29,31,37-38,41,43,45,47-48,52-54,58,61-64,66-68,71,77-78,82-86 (42)
16. from 15 keep 4,8-10,12-15,17-19,23,26-28 (15)

Table S1. Newcastle-Ottawa quality assessments of the included cohort/cross-sectional studies.

Sources (Study design)	Selection: Representativeness of the exposed cohort	Selection: Selection of the non-exposed cohort	Selection: Ascertainment of exposure	Selection: Demonstration that outcome of interest was not present at start of study	Comparability: Comparability of cohorts on the basis of the design or analysis	Outcome assessment: Assessment of outcome	Outcome assessment: Was follow-up long enough for outcomes to occur	Outcome assessment: Adequacy of follow up of cohorts	Total Quality Score
Fei et al. 2013 (PCO)	No	Yes	Yes	Yes	Yes	Yes	Yes	Yes	7/9
Guan et al. 2012 (CS)	No	No	Yes	NA	Yes	Yes	NA	NA	3/9
Cherry et al. 2010 (RCO)	Yes	Yes	Yes	Yes	Yes	Yes	Yes	NA	7/9
Myers et al. 2010 (RCO)	Yes	Yes	No	Yes	Yes	Yes	Yes	Yes	7/9
Rahman et al. 2010 (PCO)	Yes	Yes	Yes	Yes	No	Yes	Yes	Yes	7/9
Rahman et al. 2009 (PCO)	Yes	Yes	Yes	Yes	Yes	Yes	Yes	Yes	9/9
Cherry et al. 2008 (RCO)	Yes	Yes	No	Yes	Yes, strong	Yes	Yes	Yes	8/9
Sen and Chaudhuri 2008 (CS)	Yes	Yes	No	NA	No	NA	NA	NA	2/9
Huyck et al. 2007 (PCO)	No	Yes	Yes	Yes	Yes	Yes	Yes	Yes	7/9
Rahman et al. 2007 (PCO)	Yes	Yes	Yes	Yes	Yes	Yes	Yes	NA	7/9
Ahamed et al. 2006 (CS)	No	Yes	No	NA	No	NA	NA	NA	1/9
von Ehrenstein et al. 2006 (CS)	Yes	Yes	No	NA	Yes	NA	NA	NA	3/9
Milton et al. 2005 (CS)	Yes	Yes	No	NA	Yes	NA	NA	NA	3/9
Mukherjee et al. 2005 (CS)	No	Yes	No	NA	No	NA	NA	NA	1/9
Rahman et al. 2005 (CS)	No	Yes	No	NA	No	NA	NA	NA	1/9
Chakraborti et al 2003 (CS)	No	Yes	No	NA	No	NA	NA	NA	1/9
Guo et al. 2003 (CS)	Yes	Yes	No	NA	Yes	NA	NA	NA	3/9
Hopenhayn et al. 2003 (PCO)	Yes	Yes	No	Yes	Yes	Yes	Yes	No	6/9
Yang et al. 2003 (RCO)	Yes	Yes	No	Yes	Yes	Yes	Yes	No	6/9
Ahmad et al. 2001 (CS)	Yes	Yes	No	NA	No	NA	NA	NA	2/9
Hopenhayn-Rich et al. 2000 (RCO)	Yes	Yes	No	Yes	Yes	Yes	Yes	No	6/9

NA: Not applicable. PCOS: Prospective cohort study, RCOS: retrospective cohort study, CS: cross-sectional study.

Table S2. Newcastle-Ottawa quality assessments of the included case-control studies.

Sources (Study design)	Selection: Is the case definition adequate?	Selection: Representativeness of the cases	Selection: Selection of Controls	Selection: Definition of Controls	Comparability: Comparability of cases and controls on the basis of the design or analysis	Exposure assessment: Ascertainment of exposure	Exposure assessment: Same method of ascertainment for cases and controls	Exposure assessment: Non-response rate	Total Quality Score
Ihrig et al. 1998 (C-C)	Yes	Yes	Yes	Yes	Yes	No	Yes	Yes	7/9
Aschengrau et al. 1989 (C-C)	Yes	Yes	Yes	Yes	Yes	No	Yes	Yes	7/9

CC: Case-control study.

Table S3. Core and additional confounders for spontaneous abortion, stillbirth, birthweight/low birth weight, preterm delivery and infant/neonatal death documented in the literature.

Endpoint	Core confounders[a]	Additional confounders[b]
Spontaneous abortion and stillbirth	maternal age, tobacco smoke, and previous history of spontaneous abortion	Socio-economic status, education, marital status, other sociodemographic variables
Birth weight/low birth weight and preterm delivery	maternal age, tobacco smoke, pre-pregnancy weight (or pre-pregnancy BMI or other indicators of maternal nutrition)	Socio-economic status, education, marital status, other sociodemographic variables
Neonatal/infant death	antenatal care, maternal nutrition (pre-pregnancy BMI, pre-pregnancy weight, height), socio-economic status	Maternal age, marital status, other sociodemographic variables

[a]A known potential confounder of the association between arsenic and the outcome of interest. [b]A factor that may confound the association between arsenic and the outcome of interest.

Table S4. Studies on arsenic and spontaneous abortion (n=6), stillbirth (n=9), preterm delivery (n=3), and birth weight (n=4) included in the meta-analysis, and point estimates (95% confidence intervals) for each outcome.

Sources	Arsenic marker for exposure	Arsenic exposure contrast	Spontaneous abortion	Still birth	Preterm delivery	Birth weight in grams
Myers et al. 2010[a]	arsenic levels in tube well water	>50 µg/L vs. ≤50 µg/L		OR=2.01 (1.12, 3.59)	OR=1.02 (0.72, 1.44)	
Rahman et al. 2010[a]	Arsenic levels in urine	249-1253 µg/L vs. <33 µg/L	OR=1.44 (0.96, 2.15)	NA	N/A	N/A
Rahman et al. 2010[a]	Arsenic levels in urine	268-2019 µg/L vs. <38 µg/L	NA	OR=2.02 (0.50, 8.20)	NA	NA
Rahman et al. 200[a]9	Arsenic concentration in urine	≥100 µg/L vs. <100 µg/L	N/A	N/A	N/A	-82.42 (-167.56, -2.72)
Cherry et al. 2008[a]	Average arsenic concentrations in hand pump well water	≥50 µg/L vs. <0.10 µg/L	N/A	OR=1.20 (0.97, 1.29)	N/A	N/A
Rahman et al. 2007[a]	Arsenic levels in tube-well water	≥409 µg/L vs. <10 µg/L	N/A	RR=1.12 (0.97, 1.29)	N/A	N/A
Huyck et al. 2007[a]	Arsenic levels in maternal hair at first prenatal visit	≥2.70 µg/g vs. <0.09 µg/g	N/A	N/A	N/A	-193.5 (-369.9, -17.10)
von Ehrenstein et al. 2006[a]	Arsenic level in tube-well water	≥200 µg/L vs. <50 µg/L	OR=1.01 (0.38, 2.70)	OR=6.07 (1.54, 23.96)	N/A	N/A
Milton et al. 2005[a]	Arsenic levels in tube-well water	>50 µg/L vs. ≤50 µg/L	OR=2.5 (1.5, 4.23)	OR=2.5 (1.29, 4.85)	N/A	N/A
Guo et al. 2003[b]	Arsenic level in well water	Exposed area (43 µg/L) vs. non-exposed area (9.6 µg/L	RR=2.7 (0.83, 8.75)	N/A	N/A	N/A
Hopenhayn et al. 2003[b]	Arsenic level in water	(32.9-52.7) µg/L vs. (0.5-1.1) µg/L	N/A	N/A	N/A	-57 (-122.99, 8.99)
Yang et al. 2003[a]	High exposed community used as a surrogate	Exposed area (3590µg/L) vs. non-exposed area	N/A	N/A	OR=1.10 (0.91,1.33)	-29.05 (-44.55, -13.55)
Ahmad et al. 2001[a]	Arsenic level in tube-well water	>50 µg/L vs. ≤20 µg/L	OR=2.90 (2.20, 3.82)	OR=2.24 (1.55,3.24)	OR=2.54 (1.85,3.49)	N/A
Hopenhayn-Rich et al. 2000[a]	Arsenic level in public water	>50 µg/L vs. 5 µg/L	N/A	RR=1.81 (1.58, 2.08)	N/A	N/A
Ihrig et al. 1998[a]	Arsenic level estimate from airborne emissions	>100 ng/m^3 vs. 0 ng/m^3	N/A	OR=4.0 (1.80,13.54)	N/A	N/A
Aschengrau et al. 1989[b]	arsenic level in public drinking water	(1.4-1.9) µg/L vs. undetectable limit	OR=1.5 (0.44, 4.78)	N/A	N/A	N/A

Abbreviations: n, number of studies; N/A, not available.

[a]Studies measuring moderate-to-high arsenic exposure levels. [b]Studies measuring low-to-moderate arsenic exposure levels.

Table S5. Studies on arsenic and neonatal (n=5) and infant mortality (n=7) included in the meta-analysis and point estimates (95% confidence intervals) for each outcome.

Source	Marker for arsenic exposure	Exposure contrast	Neonatal mortality	Infant mortality
Cherry et al. 2010[a]	Arsenic levels in tube-well water	≥ 50 μg/L vs. <10 μg/L	N/A	OR=1.20 (0.90, 1.59)
Myers et al. 2010[a]	Arsenic levels in tube well water	>50 μg/L vs. ≤50 μg/L	OR=2.01 (1.12, 3.59)	OR=2.01 (1.12, 3.59)
Rahman et al. 2010[a]	Arsenic levels in urine	268-2019 μg/L vs. <38 μg/L	N/A	OR=5.01 (1.41, 17.82)
Rahman et al. 2007[a]	Arsenic levels in tube-well water	≥409μg/L vs. <10 μg/L	RR=1.23 (0.97, 1.56)	RR=1.19 (1.00, 1.41)
von Ehrenstein et al. 2006[a]	Arsenic levels in tube-well water	≥200μg/L vs. <50 μg/L	OR=2.81 (0.73, 10.81)	OR=1.33 (0.43, 4.12)
Milton et al. 2005[a]	Arsenic levels in tube-well water	>50μg/L vs. ≤50 μg/L	OR=1.8 (0.91, 3.57)	OR=1.80 (0.91, 3.55)
Hopenhayn-Rich et al. 2000[a]	Arsenic levels in public water	>50 μg/L vs. 5μg/L	RR=1.70 (1.40, 1.76)	RR=1.30 (94, 1.80)

Abbreviations: n, number of studies; N/A, not available.

[a]Studies measuring moderate-to-high arsenic exposure levels.

Table S6. Studies excluded from the systematic review and meta-analysis.

Source	Reasons for exclusion
Börzsönyi et al. 1992	A short commentary
Brender et al. 2006	Outcome definition not compatible with ours
Chen et al. 2009	Exposure definition not compatible with ours
Cherry et al. 2012	Exposure definition not compatible with ours
Hafeman et al. 2007	Exposure definition not compatible with ours
Hamadani et al. 2011	Outcome definition not compatible with ours
Huang et al. 2011	Exposure definition not compatible with ours
Jin et al. 2013	Outcome definition not compatible with ours
Kippler et al. 2012	Outcome definition not compatible with ours
Kippler et al. 2012	Exposure definition not compatible with ours
Landgren 1996	Arsenic levels in small streams not related with human exposure
Llanos and Ronco 2009	Outcome definition not compatible with ours
Nordenson et al. 1978a	Applied job title as a proxy for arsenic exposure
Nordenson et al. 1978b	Applied living near a smelting house as a proxy for arsenic exposure
Nordstrom et al .1978a	Applied job title as a proxy for arsenic exposure
Nordstrom et al. 1978b	Applied living near a smelting house as a proxy for arsenic exposure
Rahman et al. 2011	Outcome definition not compatible with ours
Raqib et al. 2009	Outcome definition not compatible with ours
Saha et al. 2012	Outcome definition not compatible with ours
Mukherjee et al 2006	Overlaps with Mukherjee et al 2005
Shirai et a.l 2010	Reported arsenic in sea-foods
Sohel et al.. 2009	Outcome definition not compatible with ours
Thakur et al. 2010	Outcome definition not compatible with ours
Tofail et al. 2009	Outcome definition not compatible with ours
Tollestrup et al. 2003	Outcome definition not compatible with ours
Tsai et al. 1999	Outcome definition not compatible with ours
Wade et al. 2009	Outcome definition not compatible with ours
Wu et al. 2011	Outcome definition not compatible with ours
Wulff et al. 2002	Applied job title as a proxy for arsenic exposure
Wulff et al. 1996	Applied living near a smelting house as a proxy for arsenic exposure
Wulff et al. 1995	Applied job title as a proxy for arsenic exposure
Yu and Zhang 2011	Definition of exposure compatible with ours
Zieler et al. 1988	Outcome definition not compatible with ours

References

Börzsönyi M, Bereczky A, Rudnai P, Csanady M, Horvath A. 1992. Epidemiological studies on human subjects exposed to arsenic in drinking water in southeast Hungary. Arch Toxicol 66:77-78.

Brender JD, Suarez L, Felkner M, Gilani Z, Stinchcomb D, Moody K, et al. 2006. Maternal exposure to arsenic, cadmium, lead, and mercury and neural tube defects in offspring. Environ Res 101:132-139.

Chen G, Pei LJ, Huang J, Song XM, Lin LM, Gu X et al. 2009. Unusual patterns of neural tube defects in a high risk region of northern China. Biomed Environ Sci 22:340-344.

Cherry N, McDonald C, Chowdhury Z. 2012. Zinc in well water and infant mortality in bangladesh: a report from gonoshasthaya kendra. Int J Environ Res Public Health 9:171-177.

Hafeman D, Factor-Litvak P, Cheng Z, van Geen A, Ahsan H. 2007. Association between manganese exposure through drinking water and infant mortality in Bangladesh. Environ Health Perspect 115:1107-1112.

Hamadani JD, Tofail F, Nermell B, Gardner R, Shiraji S, Bottai M, et al. 2011. Critical windows of exposure for arsenic-associated impairment of cognitive function in pre-school girls and boys: a population-based cohort study. Int J Epidemiol 40:1593-1604.

Huang J, Wu J, Li T, Song X, Zhang B, Zhang P, Zheng X. 2011. Effect of exposure to trace elements in the soil on the prevalence of neural tube defects in a high-risk area of China. Biomed Environ Sci 24:94-101.

Jin L, Zhang L, Li Z, Liu JM, Ye R, Ren A. 2013. Placental concentrations of mercury, lead, cadmium, and arsenic and the risk of neural tube defects in a Chinese population. Reprod Toxicol 35:25-31.

Kippler M, Wagatsuma Y, Rahman A, Nermell B, Persson LÅ, Raqib R et al. 2012. Environmental exposure to arsenic and cadmium during pregnancy and fetal size: a longitudinal study in rural Bangladesh. Reprod Toxicol 34:504-511.

Kippler M, Tofail F, Gardner R, Rahman A, Hamadani JD, Bottai M et al. 2012. Maternal cadmium exposure during pregnancy and size at birth: a prospective cohort study. Environ Health Perspect 120:284-289.

Landgren O. 1996. Environmental pollution and delivery outcome in southern Sweden: a study with central registries. Acta Paediatr 85:1361-1364.

Llanos MN, Ronco AM. 2009. Fetal growth restriction is related to placental levels of cadmium, lead and arsenic but not with antioxidant activities. Reprod Toxicol 27:88-92.

Nordenson I, Beckman G, Beckman L, Nordström S. 1978a. Occupational and environmental risks in and around a smelter in northern Sweden. IV. Chromosomal aberrations in workers exposed to lead. Hereditas 88:263-267.

Nordenson I, Beckman G, Beckman L, Nordström S. 1978b. Occupational and environmental risks in and around a smelter in northern Sweden. II. Chromosomal aberrations in workers exposed to arsenic. Hereditas 88:47-50.

Nordström S, Beckman L, Nordenson I. 1978a. Occupational and environmental risks in and around a smelter in northern Sweden. III. Frequencies of spontaneous abortion. Hereditas 88:51-54.

Nordström S, Beckman L, Nordenson I. 1978b. Occupational and environmental risks in and around a smelter in northern Sweden. I. Variations in birth weight. Hereditas 88:43-46.

Rahman A, Vahter M, Ekström EC, Persson LÅ. 2011. Arsenic exposure in pregnancy increases the risk of lower respiratory tract infection and diarrhea during infancy in Bangladesh. Environ Health Perspect 119:719-724.

Raqib R, Ahmed S, Sultana R, Wagatsuma Y, Mondal D, Hoque AM, et al. 2009. Effects of in utero arsenic exposure on child immunity and morbidity in rural Bangladesh. Toxicol Lett 185:197-202.

Saha KK, Engström A, Hamadani JD, Tofail F, Rasmussen KM, Vahter M. 2012. Pre- and postnatal arsenic exposure and body size to 2 years of age: a cohort study in rural Bangladesh. Environ Health Perspect 120:1208-1214.

Mukherjee A, Sengupta MK, Hossain MA, Ahamed S, Das B, Nayak B, Lodh D, Rahman MM, Chakraborti D. 2006. Arsenic contamination in groundwater: a global perspective with emphasis on the Asian scenario. J Health Popul Nutr. 2006 Jun;24(2):142-63.

Shirai S, Suzuki Y, Yoshinaga J, Mizumoto Y. 2010. Maternal exposure to low-level heavy metals during pregnancy and birth size. J Environ Sci Health A Tox Hazard Subst Environ Eng 45:1468-1474.

Sohel N, Persson LA, Rahman M, Streatfield PK, Yunus M, Ekström EC et al. 2009. Arsenic in drinking water and adult mortality: a population-based cohort study in rural Bangladesh. Epidemiology 20:824-830

Thakur JS, Prinja S, Singh D, Rajwanshi A, Prasad R, Parwana HK, et al. 2010. Adverse reproductive and child health outcomes among people living near highly toxic waste water drains in Punjab, India. J Epidemiol Commun Health 64:148-154.

Tofail F, Vahter M, Hamadani JD, Nermell B, Huda SN, Yunus M et al.2009. Effect of arsenic exposure during pregnancy on infant development at 7 months in rural Matlab, Bangladesh. Environ Health Perspect 117:288-293.

Tollestrup K, Frost FJ, Harter LC, McMillan GP. 2003. Mortality among children residing near the American Smelting and Refining Company (ASARCO) copper smelter in Ruston, Washington. Arch Environ Health 58:683-691.

Tsai SM, Wang TN, Ko YC. 1999. Mortality for certain diseases in areas with high levels of arsenic in drinking water. Arch Environ Health 54:186-193.

Wade TJ, Xia Y, Wu K, Li Y, Ning Z, Le XC, et al. 2009. Increased mortality associated with well-water arsenic exposure in Inner Mongolia, China. Int J Environ Res Public Health 6:1107-1123.

Wu J, Chen G, Liao Y 2011. Arsenic levels in the soil and risk of birth defects: a population-based case-control study using GIS technology. J Environ Health 74:20-25.

Wulff M, Högberg U, Stenlund H. 2002. Occupational and environmental risks of spontaneous abortions around a smelter. Am J Ind Med 41:131-138.

Wulff M, Högberg U, Sandström-Holmgren A. 1996. Congenital malformations in the vicinity of a smelter in Northern Sweden, 1973-1990. Paediatr Perinat Epidemiol 10:22-31.

Wulff M, Högberg U, Sandström AI. 1995. Perinatal outcome among the offspring of employees and people living around a Swedish smelter. Scand J Work Environ Health 21:277-282.

Yu HY, Zhang KL. 2011. Links between environmental geochemistry and rate of birth defects: Shanxi Province, China. Sci Total Environ 409:447-479

17:589-594.

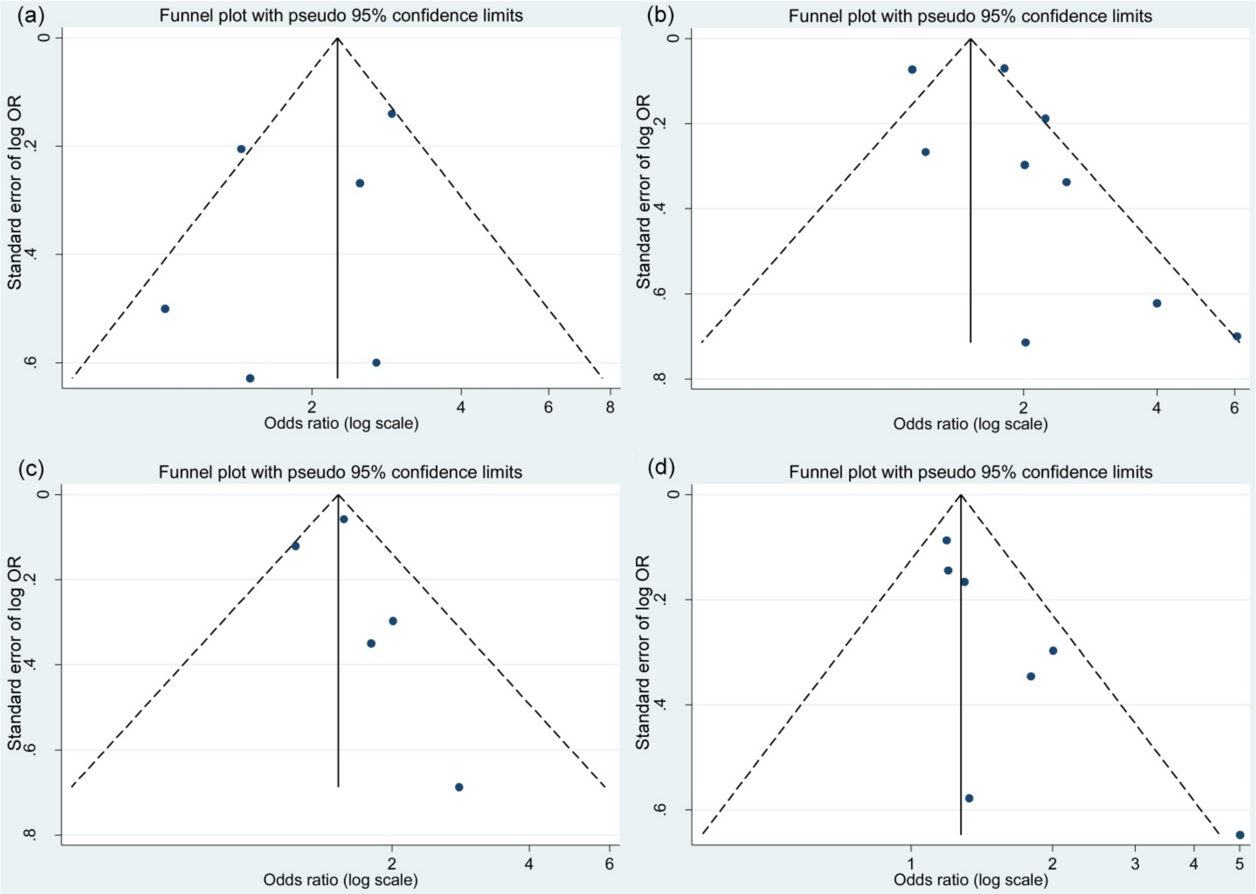

Figure S1. Funnel plots for the relation between arsenic and (a) spontaneous abortion, (b) stillbirth, (c) neonatal mortality and (d) infant mortality.
